# The translational factor eIF3f: the ambivalent eIF3 subunit

**DOI:** 10.1007/s00018-013-1263-y

**Published:** 2013-01-25

**Authors:** Roberta Marchione, Serge A. Leibovitch, Jean-Luc Lenormand

**Affiliations:** 1 HumProTher Laboratory, TheREx, TIMC-IMAG Laboratory, CNRS UMR5525, University Joseph Fourier, 38700 La Tronche, Cedex, France; 2 Laboratoire de Génomique Fonctionnelle et Myogenèse, UMR 866 DMEM, INRA UM II, Campus INRA/Sup Agro, 2 Place Pierre Viala, 34060 Montpellier, Cedex 1, France

**Keywords:** Apoptosis, Atrophy, Cancer, eIF3f, Hypertrophy

## Abstract

The regulation of the protein synthesis has a crucial role in governing the eukaryotic cell growth. Subtle changes of proteins involved in the translation process may alter the rate of the protein synthesis and modify the cell fate by shifting the balance from normal status into a tumoral or apoptotic one. The largest eukaryotic initiation factor involved in translation regulation is eIF3. Amongst the 13 factors constituting eIF3, the f subunit finely regulates this balance in a cell-type-specific manner. Loss of this factor causes malignancy in several cells, and atrophy in normal muscle cells. The intracellular interacting partners which influence its physiological significance in both cancer and muscle cells are detailed in this review. By delineating the global interaction network of this factor and by clarifying its intracellular role, it becomes apparent that the f subunit represents a promising candidate molecule to use for biotherapeutic applications.

## Introduction

Protein synthesis is one of the most complex and energy-consuming processes in eukaryotic cells, by which the genetic information is translated from a nucleic acid code into an amino acidic language to make proteins. Global protein synthesis rates have a key role in governing cell growth, the control of cell size, and cell proliferation. Changes in the levels of specific regulatory proteins involved in the translation process may affect the translation rates and the amount of produced proteins. Thus, the regulation of gene expression is a crucial step in cellular physiology considering that subtle defects in the mechanism of translational control may lead to either cell malignancy or cell death. Protein synthesis is mainly regulated at the initiation step. In eukaryotes, initiation can be divided into two steps: formation of a 48S initiation complex and its joining with a 60S subunit [1]. First, the eukaryotic initiation factors eIF3, eIF1, eIF1A and eIF2-GTP-Met-tRNAi bind to the 40S ribosomal subunit to form a 43S preinitiation complex. This complex subsequently attaches to the 5′-proximal region of mRNAs, after which eIF4A, 4B and 4F unwind their secondary structure. The 43S complex then scans the 5′ untranslated region in the 5′–3′ direction until the initiation codon, where it stops and forms a 48S complex with codon–anticodon base-pairing through eIF5. Second, eIF5B promotes the displacement of the eukaryotic initiation factors and the subsequent joining of 48S complex with the 60S subunit [1].

The largest and more complex eukaryotic initiation factor, which plays a role in translation regulation, cell growth and cancer, is eIF3. This review is focused on the function of the f-subunit of eIF3 (eIF3f) in the translation regulation process. An analysis of its physiological significance and its intracellular interacting partners involved in translational regulation both in cancer and muscle cells is sequentially detailed. The aim is to delineate the global eIF3f interaction network and to clarify its intracellular role in order to validate eIF3f as a lead candidate to use for biotherapeutic applications.

## Eukaryotic initiation factor 3

Translation initiation in eukaryotes is an intricate process requiring at least nine eukaryotic initiation factors [1, 2]. Among these factors, eIF3 is the largest as it comprises up to 13 non-identical subunits. Several functions have been ascribed to eIF3, including the interaction and stabilization of the eIF2-GTP-Met-tRNAi ternary complex, binding to the 40S ribosomal subunit, facilitating the binding of mRNA to the 40S ribosome, and promoting dissociation of the 40S and 60S ribosomal subunits. Other initiation factors (e.g., eIF4G, eIF4B, eIF5 and eIF1) are known to interact with eIF3, suggesting that this factor might have a role in ‘organizing’ proteins on the surface of the 40S ribosome [3–5].

## Structure and nomenclature

Mammalian eIF3 is an 800-kDa molecular mass assembly of 13 subunits that are designated eIF3a–eIF3m [3–11]. In the past, ad hoc nomenclatures for eIF3 subunits have been derived from subunit molecular weights or gene names. In 2001, to eliminate confusion surrounding cross-species comparisons of eIF3 subunits, a unified nomenclature was proposed by Browning et al. [7]. Each subunit is identified by a letter designation based on the decreasing order of the mammalian subunit masses determined from SDS-PAGE mobility (Table 1). For two novel subunits, −l and −m, the nomenclature has been adapted as they have arisen.

**Table 1:** Summary of eukaryotic eIF3 subunits

Unified nomenclature	*S. cerevisiae* + MW	*H. sapiens*	*S. pombe*	*A. thaliana*	Consensus motif
**eIF3a**	**Tif32—110** **kDa**	**p170**	**p107**	**p114**	**PCI**
**eIF3b**	**Prt1—90** **kDa**	**p116**	**p84**	**p82**	**RRM**
**eIF3c**	**Nip1—93** **kDa**	**p110**	**p104**	**p105**	**PCI**
*eIF3d*	–	*p66*	*Moe1* ^*a*^	*p66*	*–*
eIF3e	–	p48	*Int6* ^*a*^	*p51*	*PCI*
eIF3f	–	p47	*Csn6*	*p32*	*MPN*
*eIF3g*	**Tif35—33** **kDa**	**p44**	**Tif35**	**p33**	**RBD**
eIF3h		p40	*p40* ^*b*^	*p38*	*MPN*
*eIF3i*	**Tif34—39** **kDa**	**p36**	**Sum1**	**p36**	**WD repeats**
*eIF3j*	*Hcr1—35* *kDa*	*p35*	*–*	*–*	*–*
*eIF3k*	*–*	*p28*	*–*	*p25*	*PCI*
*eIF3l*	*–*	*p67*	*–*	*p60*	*PCI*
*eIF3m*	*–*	*GA17*	*Csn7B* ^*b*^	*–*	*PCI*

The ‘conserved’ core subunits are highlighted in bold type, the ‘functional’ core subunits are underlined, and the ‘dispensable’ ones are in italics

*MPN* Mpr1p and Pad1p N-terminal conserved domain;* PCI* 26S proteasome, COP9 signalosome and eukaryotic initiation factor eIF3 conserved domain;* RBD* RNA-binding domain;* RRM* RNA-recognition motif;* S6K1* ribosomal protein S6 kinase 1;* WD* conserved regions of approximately 40 amino acids typically bracketed by Trp–Asp

^a^Subunits contained in *S. pombe* Int6 eIF3 complex

^b^Subunits contained in *S. pombe* Csn7B eIF3 complex

Initial characterizations of mammalian eIF3 were based on biochemical methods applied to purified proteins from rabbit reticulocytes and HeLa cells [12, 13]. Due to its large size and complexity, a detailed understanding of eIF3 structure and function has been achieved more recently from experiments based on the cloning and characterization of human cDNAs encoding the eIF3 subunits [5, 14–16]. By comparing the cDNA sequences of the mammalian eIF3 subunits to the entire genome of the budding yeast *Saccharomyces cerevisiae*, it appears that only five mammalian subunits eIF3-a, -b, -c, -g and -i have identifiable homologues encoded in yeast (Table 1). A sixth ortholog, eIF3j, is a nonessential subunit of the budding yeast eIF3 that enhances interactions with other eIFs [17], promotes binding of eIF3 to the 40S subunit [18], and has an independent function in 40S ribosome biogenesis [19]. Biochemical interactions have been detected between these five conserved subunits in both mammalian and yeast systems [14, 15, 20]. The *S. cerevisiae* eIF3 complex also contains 3 additional proteins, p135, p62 and p16, for which corresponding homologs are not found in the mammalian complex [21–23], whereas 7 subunits (eIF3-d, -e, -f, -h, -k, -l and -m) identified in the mammalian eIF3 complex are absent in the yeast complex (Table 1). Most of these 7 mammalian subunits of eIF3 appear to be highly conserved in *Drosophila melanogaster*, *Caernorhabditis elegans*, *Arabidopsis thaliana* and the fission yeast *Schizosaccharomyces pombe* [4, 5]. Nevertheless, the biochemical function of this complex is conserved inasmuch as eIF3 purified from *S. cerevisiae* can replace mammalian eIF3 in the in vitro methionyl-puromycin synthesis assay [21]. Accordingly, the mammalian eIF3 complex consists of five subunits (eIF3-a, -b, -c, -g, -i) that represent a ‘conserved’ core essential for translation initiation in vivo, and the remaining ‘noncore’ subunits that appear to be evolutionarily divergent and serve to modulate its activity [5, 14, 20].

The function of each subunit of eIF3 has first been investigated in *S. cerevisiae*. Deletion or mutation of eIF3a [24, 25], eIF3b [15, 26], eIF3c [27], eIF3g [28], or eIF3i [20, 28, 29] in yeast leads to a vast reduction in protein synthesis, suggesting that each of these five subunits is required for eIF3 integrity. The role of each subunit and the minimum combination of components required for the formation of an active and functional core have also been investigated in mammalian eIF3 [30]. By using a baculovirus-based coexpression system, recombinant eIF3 complexes lacking different individual subunits have been reconstituted. Remarkably, deletion studies showed that the rate of the protein synthesis does not change by knockdown of the two evolutionarily conserved subunits, eIF3g and eIF3i, in HeLa cells, and the eIF3 activity remains unaltered after deletion of the three non-conserved subunits eIF3-d, -k, -l [30]. Therefore, eIF3-g, -i, -d, -k, -l subunits are not essential for eIF3 activity but might be involved in translational control of specific mRNAs or in particular cellular conditions [30]. Hence, the functional core of mammalian eIF3 consists of three conserved subunits, eIF3-a, -b, -c, and three non-conserved subunits, eIF3-e, -f and -h. These three last subunits might serve to stabilize the eIF3-abc complex which directly participates in translation initiation [30]. In vitro stepwise reconstitution of human eIF3 subunits has revealed the architectural features of this factor. The dimer of subunits eIF3a and eIF3c serves as a central scaffold to which most of the other subunits bind. This dimer directly interacts with the subunits eIF3e, -k, -l and -m containing the PCI domain (the 26S proteasome, COP9 signalosome and eukaryotic initiation factor eIF3 conserved domain) and with the subunits eIF3-f and-h containing a MPN (Mpr1p and Pad1p N-terminal conserved domain) to form a highly stable PCI/MPN octamer. Subunits -d, -b, -g, -i, and -j then finalize the assembly of the eIF3 complex [11].

## eIF3 function in cell cycle regulation, translational regulation, and cancer

The global protein synthesis in mammals occurs mainly in the G1 phase of a cell cycle and is reduced during mitosis. This process is modulated by several translation initiation factors [31]. The first studies that associated two eIF3 subunits, eIF3a and eIF3b, to cell cycle regulation were performed in *S. cerevisiae*. Both subunits are essential for the G1-S phase transition in yeast [32, 33]. A study on the eIF3a expression in mammalian cells has shown that its levels oscillate during the cell cycle and its maximum of expression is detected in the S phase, indicating that eIF3a may be a translational regulator for proteins important for S phase entrance [34]. Additionally, the subunits eIF3a [35], eIF3e [36, 37], eIF3k [38] and eIF3f [39] have been found in both cytoplasm and nucleus, indicating that these factors may shuttle between the two subcellular localizations according to cell cycle progression.

Proper execution of the cell cycle requires the synthesis and activation of key proteins at specific times. The most prevalent mechanism for regulating the overall rate of protein synthesis involves the phosphorylation of the initiation factors. Besides phosphorylation, the rate of protein synthesis is regulated by other mechanisms and post-translational modifications of the translational machinery, such as methylation of lysine or arginine residues and O-glycosylation [40]. Recent reports confirm the role of the eIF3 factor in regulation of translation initiation rate [40–42]. However, the molecular mechanisms by which the function of eIF3 is regulated are poorly understood. A mass spectrometric approach has been used to determine post-translational modifications that regulate the activity of eIF3 during the translation initiation process (Table 2). A total of 29 phosphorylation sites and several other post-translational modifications (loss of N-terminal methionine and/or N-terminal protein acetylation) have recently been identified [10].

**Table 2:** Identification of eIF3 protein subunits and their corresponding post-translational modifications using a mass spectrometric analysis [10]

Protein name	UniProt accession number	Molecular mass^a^ (Da)	Sequence coverage (%)	Post-translational modifications
eIF3a	Q14152	166,758.3	86	Loss of Met-1, phosphorylation (Ser-881, Ser-1198, Ser-1336^b^, Ser-1364^b^)
eIF3b	P55884	93,093.7	77	Acetylation (Met-1), phosphorylation (Ser-83, Ser-85, Ser-119, Ser-125^b^, Ser-152, Ser-154, Ser-164)
eIF3c	Q99613	106,143.8	65	Phosphorylation (Ser-9, Ser-11, Ser-13, Ser-15, Ser-16, Ser-18, Ser-39, Ser-166^c^, Thr-524^b^, Ser-909^c^)
eIF3d	O15371	63,972.9	74	Not found
eIF3e	P60228	52,131.8	84	Loss of Met-1, acetylation (Ala-2)
eIF3f	O00303	37,554.8	79	Loss of Met-1, acetylation (Ala-2), phosphorylation (Ser-258^b^)
eIF3g	O75821	35,639.8	83	Loss of Met-1, phosphorylation (Thr-41, Ser-42)
eIF3h	O15372	40,010.4	89	Phosphorylation (Ser-183^b^)
eIF3i	Q13347	36,501.9	93	Not found
eIF3j	O75822	29,293.2	81	Loss of Met-1, acetylation (Ala-2), phosphorylation (Ser-11, Ser-13, Ser-20, Thr-109^c^)
eIF3k	Q9UBQ5	24,970.6	75	Loss of Met-1, acetylation (Ala-2)
eIF3l	Q9Y262	66,637.9	70	Loss of Met-1, acetylation (Ser-2)
eIF3m	Q7L2H7	42,413.8	74	Loss of Met-1, acetylation (Ser-2)

^a^Calculated from the theoretical average mass of the corresponding eIF3 protein subunit plus any post-translational modifications identified

^b^Found only after TiO_2_ phosphopeptide enrichment

^c^Found only after Ga(III) IMAC or TiO_2_ phosphopeptide enrichment

Alternatively, an indirect regulation of the activity of eIF3 is performed by association of its subunits with other proteins involved in the regulation of protein synthesis. For example, the subunit eIF3e binds p56 in interferon-treated or virus-infected mammalian cells, and inhibits the translation in vitro and in vivo [43, 44]. The subunit eIF3g interacts with Paip1, a Poly (A)-binding protein and stimulates translation initiation [45], whereas the subunits eIF3h and eIF3f interact with TRC8, a ubiquitin E3 ligase, and inhibit protein synthesis, possibly through ubiquitilation of eIF3 or some other translational components [46]. These mechanisms and interacting partners render eIF3 a pivotal player in controlling the protein synthesis and degradation.

A proper level of translation initiation is necessary to regulate cell proliferation, since the hyperactivation or the down-regulation of the rate of the protein synthesis contributes importantly to cell malignancy. The contribution of eIF3 to oncogenesis and maintenance of the cancer state has been demonstrated in several studies [47–49]. Amongst the 13 subunits constituting eIF3, overexpression of subunits eIF3-a, -b, -c, -h, -i, and -m has been detected in several different solid tumors and in several different cancer cell lines (Table 3; for literature citations, see [42, 47, 48]. The correlation of abnormal eIF3 subunit levels and cancer indicates that eIF3 has an important role in determining the balance between cell proliferation and apoptosis. A study performed on NIH3T3 fibroblasts, stably transfected with five individual eIF3 subunit cDNAs, has confirmed that their overexpression contributes to malignant phenotype [48]. Ectopic overexpression of eIF3-a, -b, -c, -h or -i induces an increase in protein synthesis rate, the resistance to apoptosis, and the oncogenic transformation of NIH3T3 cells. Hence, eIF3 may play a causal role in neoplasia, and the misregulation of translation may be a contributory factor to cancer pathology [48]. It has been demonstrated that the overexpression of a single eIF3 subunit causes malignant transformation and induces an increase in eIF3 activity, which in turn stimulates the translation of mRNAs involved in cell proliferation. Zhang et al. [48] have suggested that transforming subunit overexpression leads to the preferential stimulation of so-called “weak” mRNAs, which are translated more efficiently considering the abundant presence of secondary structures in their 5′-UTRs. Generally, “weak” mRNAs encode proteins involved in promoting cell growth and proliferation, such as c-myc, cyclin D1 and growth factors. Thus, the increase of “weak” mRNA products stimulates the malignant transformation of cells.

**Table 3:** Translational factor alterations in cancers

Subunit	Observed modification	Cancer association
eIF3a	Increased expression	Mouse melanoma, human breast, cervical, esophageal, lung, and gastric cancers
eIF3b	Increased expression	Human breast carcinoma
eIF3c	Increased expression	Human testicular seminomas
eIF3e	Decreased expression	Human breast and lung carcinomas
eIF3f	Decreased expression	Pancreas, vulva, ovary, breast, small intestine tumors, and melanoma
eIF3h	Increased expression	Human breast, prostate, hepatocellular carcinomas
eIF3i	Increased expression	Cadmium transformed NIH3T3 cell lines
eIF3m	Increased expression	Human colon cancer

Two other eIF3 subunits, eIF3e and eIF3f, are downregulated in human breast/lung carcinomas and melanoma/pancreatic cancer, respectively [50–54]. Low eIF3e expression is considered as a significant factor in predicting poor prognosis for nonsmall cell lung cancer [52]. It may be likely that these factors have a causal role in cancer development inasmuch as their decrease removes the restraints that block the protein synthesis and stimulates the selective translation of oncogenic proteins involved in cell proliferation [53, 54].

## The f subunit of eIF3

In 2003, Shi et al. [39] showed, for the first time, that in human cells the noncore f subunit of the mammalian eIF3 multiprotein complex acts as a negative regulator of translation. Furthermore, in mammalian cells, eIF3f inhibits protein synthesis at the translational level and not by affecting mRNA levels [55]. Translation initiation can be cap-dependent or cap-independent/IRES-dependent. It is not clearly known whether eIF3f specifically inhibits one of these two types of translation. However, the silencing of eIF3f increased both cap-dependent and IRES-dependent translation, indicating a suppressive role of eIF3f on both translation initiation mechanisms [56]. Furthermore, it has been recently demonstrated that the f subunit plays a role in the inhibition of HIV-1 replication. Overexpression of N-terminal 91 amino acids of eIF3f or full-length eIF3f drastically restricts HIV-1 replication by interfering with the 3′-end processing of HIV-1 mRNAs and reducing their nuclear and cytoplasmic levels [57, 58].

## Intracellular localization

Under normal conditions, most of the endogenous eIF3f is bound to 40S ribosomal subunit [55]. However, confocal microscopy images of human melanoma cells show that eIF3f is localized at both cytoplasmic and nuclear levels [39]. Other eIF3 subunits, such as eIF3a, eIF3e and eIF3k, have been reported to have nuclear localization [35–38]. In fact, there are at least two different types of eIF3 complexes localized in both the cytoplasm and the nucleus of cells. Hence, eIF3 is considered as a ‘dynamic’ complex, where ‘dynamic’ means that it may contain a different and variable number of subunits [59, 60]. The cytoplasmic eIF3 complex contains the subunits eIF3a-c and f, whereas the nuclear one lacks the subunits eIF3a and f [60]. What correlates this finding and the microscopy results?

It has been demonstrated that the phosphorylation of eIF3f may specifically increase its association with other eIF3 core complex subunits. In particular, during apoptosis, eIF3f is directly phosphorylated by the cyclin-dependent kinase CDK11p46 in living cells [60], and is incorporated into a micro-complex containing eIF3b and eIF3c, but not eIF3a. By cell fractionation, it has been observed that this complex precipitates with the nucleus in the insoluble fraction of the cells. Nevertheless, eIF3f can also associate in a smaller complex consisting of eIF3a, eIF3b and eIF3c in the soluble fraction of both normal and apoptotic cells [60]. Therefore, under normal conditions, the nuclear eIF3 contains eIF3b and eIF3c and, during apoptosis, the phosphorylated eIF3f joins the nuclear complex. The model currently suggested is that the translation initiation can be regulated during apoptosis by the phosphorylated eIF3f binding to different sub-fractions of the eIF3 complex [60]. This finding, coupled with the fact that eIF3f is a Mov34 family member, led Shi et al. [39] to elaborate the hypothesis that this factor may have a nuclear function in mRNA metabolism such as splicing or transport. It is also possible that the nuclear eIF3 complex has functions other than translation initiation. For example, the synthesis of ribosomes in eukaryotes takes place in the nucleus and then ribosomes are exported to the cytoplasm. Shi et al. [60] suggest that CDK11p46 may regulate the function of the nuclear eIF3 complex by phosphorylating eIF3f, considering that cyclin-dependent kinases are involved in ribosome biogenesis and nucleolar organization.

## Regulation of eIF3f activity

**Fig. 1:**
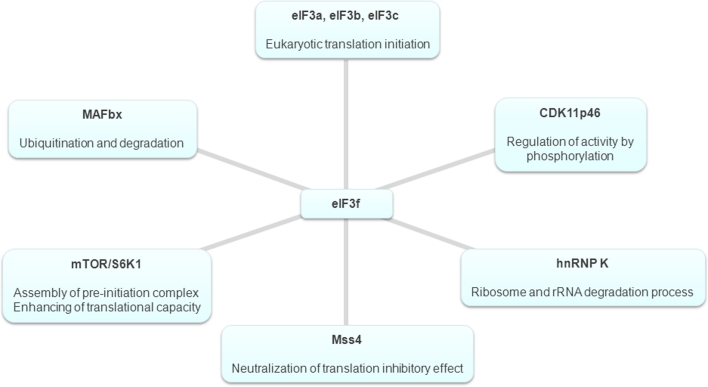
Schematic representation of the intracellular interacting partners of the eIF3f subunit

The eIF3f subunit is a Mov34 family member, containing an MPN motif, which is found in two other macromolecular complexes homologous to eIF3, the COP9 signalosome and the 19S proteasome [5, 6, 61, 62]. The presence of a MPN domain in complexes with apparently divergent functions, such as protein synthesis, signal transduction, and protein degradation, has been explained by the fact that this domain is probably necessary to promote complex assembly and is required for proper interactions between subunits of these complexes [63]. Recent crystallographic studies have revealed that there are two classes of MPN domains, one possessing a metalloprotease activity and a second possessing a structural function [64, 65]. The subunits eIF3f and eIF3h contain the second type of MPN domain, which is responsible for protein–protein interactions and for protein stability [64, 65]. The crucial role of this domain in determining the cellular function and the intracellular behaviour of eIF3f has been confirmed by several studies. In 2006, Shi et al. [55] showed that this domain, and more precisely the amino acid region spanning residues 170–248, is responsible for the translation inhibition function of eIF3f. Furthermore, it has been demonstrated that an eIF3f activating phosphorylation site is located in the Mov34 domain [60]. The phosphorylation status of this subunit can significantly influence its association with eIF3 core complex, its intracellular localization, and its function in the regulation of protein synthesis rate and in the control of the subtle balance between cell growth and cell death (see below). Moreover, several intracellular interacting partners of eIF3f have been identified (Fig. 1) using yeast two-hybrid screens. In most identified partners, the interactions are mediated by the Mov domain, suggesting that it may play a central role in regulating the activity of this subunit.

## Apoptosis versus cell growth: the importance of eIF3f rate

### Phosphorylation of eIF3f during apoptosis

Using a yeast two-hybrid system, Shi et al. [39] showed that endogenous cyclin-dependent kinase 11 (CDK11) and eIF3f protein can interact and that their interaction can be strengthened by the stimulation of apoptosis.

The CDK11 kinases belong to a large family of p34cdc2-related kinases [66] containing at least 20 CDK11 isoforms that are differentially expressed in mammalian tissues and regulate diverse cellular functions. The largest CDK11p110 isoform is associated with cyclin L and various splicing factors, and is involved in the regulation of transcription and RNA splicing in proliferating cells [67, 68]. Expression of the CDK11p110 is ubiquitous and constant throughout the cell cycle [69]. During Fas- and tumor necrosis factor-induced cell death, CDK11p110 is activated by proteolytic cleavage by caspase-3. Caspase-3 processing removes the N-terminal part of the protein, which contains nuclear translocation signals and is responsible for protein stability, and generates a smaller 46- to 50-kDa protein (CDK11p46) containing the catalytic kinase domain [70, 71].

**Fig. 2:**
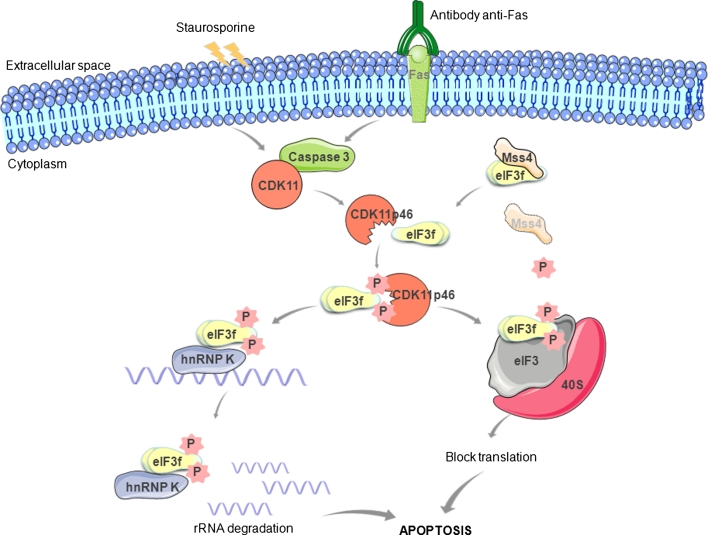
Schematic representation of eIF3f interactions during apoptosis. Upon apoptotic stimulation, CDK11p110 is cleaved by caspase 3 to generate a CDKp46 that interacts strongly with the Mov34 domain of eIF3f and phosphorylates it at Ser46 and Thr119. According to Wen et al.’s [56] hypothesis (*left side*), phosphorylated eIF3f interacts with hnRNP K and promotes rRNA degradation by interfering with rRNA protective function of hnRNP. According to Walter et al.’s [73] hypothesis, under normal conditions (*right side*), Mss4 is tightly bound to eIF3f, inhibiting its phosphorylation and subsequent association with eIF3 protein complex and pro-apoptotic functions. After prolonged stress-induced apoptosis, Mss4 is downregulated, leading to a release of eIF3f, which is phosphorylated by the CDK11p46 kinase and the translation initiation results inhibited

After anti-Fas or staurosporine treatments in human melanoma cells, the caspase-processed C-terminal fragment CDK11p46 strongly interacts with eIF3f via its Mov34 domain [39] and, due to its kinase activity, phosphorylates eIF3f inducing the inhibition of translation (Fig. 2). The mutation of the phosphate transfer site in CDK11p46 abrogates the phosphorylation of eIF3f protein. Two different phosphorylation sites have been identified in eIF3f during apoptosis: Ser46 [39] and Thr119 [60]. CDK11p46 phosphorylates both sites. Thr119 is located in the Mov34 domain of eIF3f, which is important for both the translational inhibitory function and the eIF3f interaction with CDK11p46. The biological consequence of the phosphorylation of eIF3f during apoptosis is not completely known. The phosphorylation status of eIF3f can significantly influence its association with the eIF3 core complex and its function in the regulation of translation and apoptosis [60]. Considering that the phosphorylations on Ser46 and Thr119 have been detected in cells undergoing apoptosis, it has been postulated that eIF3f may be involved in the inhibition of the protein synthesis when phosphorylated, acting as a downstream death executer.

### eIF3f expression levels in cancer and apoptotic cells

The biological role of eIF3f in translation and apoptosis in tumor cells has been investigated by Shi et al. [39, 53–56, 60]. Transcriptome analysis has shown that endogenous eIF3f expression level changes from one cell type to another and from normal to tumor cells [55]. Furthermore, the eIF3f protein level also increases during apoptosis with maximal expression at 36 h after stimulation [55].

Using a cancer profiling array and real-time reverse transcription PCR, eIF3f transcript levels have been shown to be downregulated in most human tumors relative to matched normal tissues [55]. In particular, 100 % of pancreas and vulva tumors, 90 % of breast tumors, 71 % of melanomas, and 70 % of ovary and small intestine tumors showed a significant decrease of eIF3f expression [55]. Using loss of heterozygosity and gene copy number analyses, it has been demonstrated that there is an allelic loss of *eIF3f* gene in human melanoma and in pancreatic cancer cells [53, 54]. No mutations are responsible for the decreased eIF3f expression in these two cancer types [53, 54].

The molecular mechanism by which eIF3f protein expression decreases, contributing to cancer development, is unclear. One possible explanation is that the decrease of a negative regulator of translation like eIF3f may lead to an increased eIF3 activity, which in turn stimulates translation of specific mRNAs encoding for proteins involved in cell proliferation [53, 54]. eIF3f was also found to interact with mTOR and S6K1. The decrease of eIF3f expression may deregulate the function of the mTOR pathway, which in turn leads to increased translation of oncogenic proteins and malignant transformation [53, 54]. Finally, eIF3f is ubiquitously expressed in all tissues and is associated with both subpolysomal particles and polysomes. eIF3f is localized at both cytoplasmic and nuclear levels according to the cell cycle phase and is able to associate to different sub-fractions of the eIF3 complex. Probably, these various multiproteins complexes are implicated in the role of eIF3f in discriminating the mRNAs to be translated into normal cells, and may also reflect the deregulation of the translational pathway in tumor cells.

### eIF3f overexpression in cancer inducing apoptosis

Enforced expression of eIF3f inhibits translation, cellular growth and proliferation, and induces apoptosis in melanoma and pancreatic cancer cells [53–55]. Ectopic expression of eIF3e and eIF3f subunits in stably transfected NIH3T3 fibroblasts also inhibits cell growth and proliferation [48]. Western blot analyses have demonstrated that eIF3f induces apoptosis in caspase 3/7 and in a Bcl-2-, Bax- or Bcl-XL-independent manner [55]. It is still unclear whether decreased eIF3f expression is the cause, rather than the consequence, of malignant transformation. Knockdown of endogenous eIF3f by siRNA attenuates apoptosis in melanoma cells after treatment with staurosporine as apoptotic agent [55]. Wen et al. [56] have stably knocked down endogenous eIF3f in normal human pancreatic ductal epithelial cells (HDPE) and have observed increased cell proliferation, clonogenicity, apoptotic resistance, survival, resistance to chemotherapy drug, mesenchymal morphology, and migration. eIF3f-silenced cells also show an increased cell size, nuclear pleiomorphism, aneuploidy, and cell cycle abnormality. By using an ex vivo 3D-cell culture system, they have also shown that eIF3f-silenced HPDE cells form more irregular masses with abnormal architecture and polarity (recapitulating malignant tumors in vivo), while control cells develop into a single-layer epithelial hollow spheres (resembling normal pancreatic ductal structure in vivo). eIF3f-silenced HPDE cells proliferate in an anchorage-independent manner. These observations confirm that eIF3f is an important negative regulator of cell growth and proliferation, and the decrease of its expression contributes to tumor cells’ evading apoptosis via upregulation of protein synthesis [56].

### A link between translation initiation and rRNA degradation

Besides the hypotheses described above to explain how decreased eIF3f may contribute to cancer development, a further model linking the translation initiation factor and rRNA degradation has been proposed by Wen et al. [56]. This model finds its basis in the observation that eIF3f-transfected cancer cells show a significant decrease of 60S and 80S ribosomes and 60S/40S ribosomal subunit ratio, and an increase in RNA absorbance during apoptosis [55]. It is still unclear whether the reduced 60S ribosomal subunit contributes to or is a result of apoptosis. The increasing of the absorbance is correlated to an increased amount of rRNA degradation, which contributes to the apoptotic process [55]. The major rRNA degraded in eIF3f-transfected cells is the 28S rRNA followed by the 18S rRNA in a minor rate [55]. How exactly eIF3f induces rRNA degradation is unclear. However, as shown for other eIF3 subunits, the translation may be inhibited by specific eIF3f binding to RNAs and their consequent degradation [29]. The discovery that eIF3f can interact with the heterogenous nuclear ribonucleoprotein K (hnRNP K) during apoptosis in melanoma and HPDE cells corroborates this model [56]. hnRNP K is a RNA-binding protein responsible for the maintenance of RNA stability by binding to the 3′-UTR of the mRNAs. Yeast three-hybrid screens and RNA pull-down assays have shown that hnRNP binds to 18S and 28S rRNA [72]. In human cells, dissociation of hnRNP from rRNA contributes to rRNA degradation during apoptosis [56]. However, whether hnRNP K regulates rRNA degradation is not known. Considering that hnRNP K protects rRNA from degradation whereas eIF3f promotes this process, Wen et al. [56] have proposed that eIF3f may interact with hnRNP K during apoptosis and promote rRNA degradation by interfering with its rRNA protective function (Fig. 2). In other words, eIF3f sequesters hnRNP K and inhibits its binding to rRNA, which leads to increased rRNA degradation and attenuated translation.

The rRNA synthesis and degradation in normal cells needs to be perfectly balanced in order to maintain homeostasis of protein synthesis. Under physiological conditions, eIF3f competes with rRNA for hnRNP K binding. This contributes to the maintenance of the homeostasis of rRNA level and translation in cells. Increased eIF3f expression contributes to apoptosis via hnRNP K sequestration and increased rRNA degradation. Loss of eIF3f contributes to tumorigenesis via the increased binding of hnRNP K to rRNA and an increased rRNA level. Currently, it is still unclear where and how normal human rRNA is degraded during various physiologic or pathologic cellular processes; however, eIF3f might play a role in mRNA and rRNA degradation.

### Mss4: the neutralizing agent of eIF3f translation inhibitory effect

Starting from an investigation into the regulatory properties of the mammalian suppressor of Sec4 (Mss4) in response to cellular stress, Walter et al. [73] have recently demonstrated that this protein can act as an anti-apoptotic agent by direct binding to eIF3f. High expression levels of Mss4 protein are beneficial for cells, as they protect them from stress-induced apoptosis. The expression of Mss4 is strongly regulated by stress stimuli at both transcription and post-transcriptional levels: it is upregulated at early stages of stress stimulation, but it declines with prolonged stimulation. Yeast two-hybrid screens have indicated that the most abundant Mss4 interaction partner is eIF3f, and studies on A7 melanoma cells have shown that Mss4 neutralizes the translation inhibitory effect of eIF3f [73]. Thus, Mss4 is probably involved in the regulation of protein translation, and its association to eIF3f protects cells from apoptosis. In other words, eIF3f is a negative regulator of translation: its overexpression makes apoptosis easier, whereas its downregulation has the opposite effect. Under normal conditions, Mss4 is tightly bound to eIF3f, inhibiting its phosphorylation and subsequent association with eIF3 complex. At early stages of stress stimulation, increasing amounts of Mss4 protein efficiently bind the eIF3f preventing its interaction with CDK11p46. When Mss4 proteins in cells drop-down owing to ongoing stress, the CDK11p46 can phosphorylate the released eIF3f. The translation initiation is inhibited and apoptosis is activated (Fig. 2).

## Atrophy versus hypertrophy: the importance of eIF3f

### Hypertrophy

In muscle cells, the regulation of protein synthesis rate also has an important role in the control of cell growth. Muscular hypertrophy and atrophy are two opposite and mechanistically linked phenomena regulating muscle cell size, finely determined throughout a balance between new protein accumulation and degradation of pre-existing proteins [74]. A major mediator of skeletal muscle hypertrophy is the serine/threonine-specific protein kinase Akt1. Two major downstream branches of the Akt pathway, which are relevant to muscle hypertrophy, are the mTOR pathway, which is activated by Akt, and glycogen synthase kinase 3β, which is blocked by Akt. Both pathways control the protein synthesis [74].

**Fig. 3:**
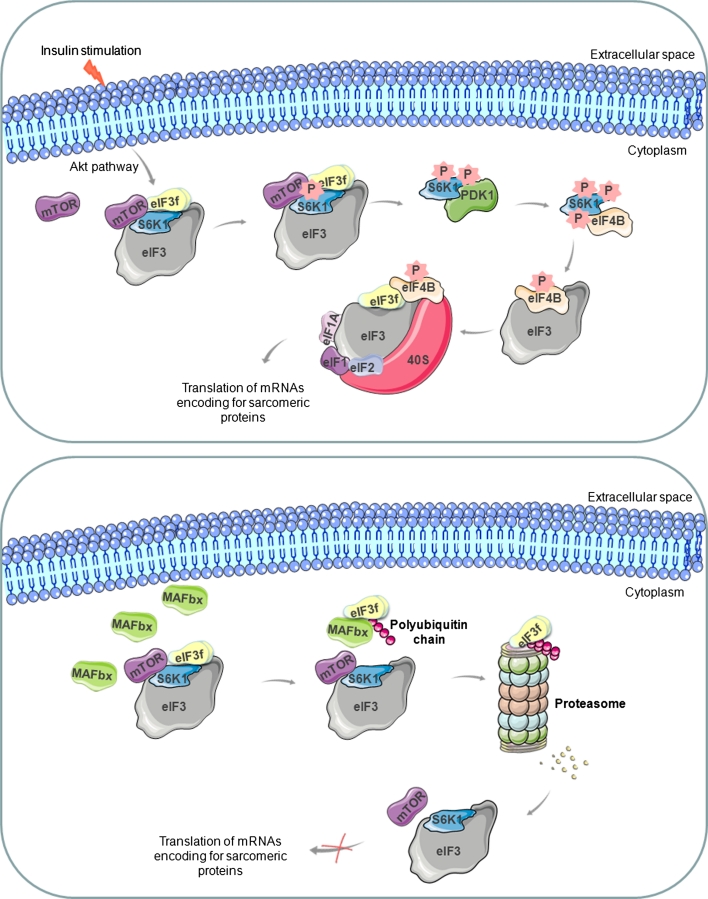
Schematic representation of the intracellular signals characterizing the hypertrophic (*upper panel*) and atrophic (*lower panel*) pathways in muscle cells. During hypertrophy, mTOR phosphorylates S6K1, which is released from eIF3 complex, and phosphorylated again by PDK1. Activated S6K1 phosphorylates eIF4B, which promotes the recruitment of other initiation factors and the translation of mRNAs encoding proteins involved in muscle growth. During atrophy, the ubiquitin ligase MAFbx is upregulated and S6K1 is accumulated in its inactive hypophosphorylated form. By binding the Mov34 domain, MAFbx transfers polyubiquitin chains on eIF3f and promotes its degradation by the proteasome and block the synthesis of proteins involved in muscle cell growth

The kinase mammalian target of rapamycin (mTOR) has recently emerged as a key regulator of mammalian cell growth that integrates signals from growth factors, nutrients, and energy status to control protein synthesis and other cell functions [75, 76]. As the name implies, mTOR is selectively inhibited by rapamycin, a drug used as an immunosuppressant in organ transplantation. The role of mTOR in muscle growth has been demonstrated by in vivo studies showing that rapamycin blocks overload hypertrophy and regenerating muscle growth [77, 78]. The effect of mTOR on the translation machinery and protein synthesis is executed by phosphorylation of the ribosomal protein S6 kinases and of 4E-BP1, a repressor of the cap-binding protein eIF4E [79, 80]. mTOR and S6K1 maneuver on and off the eIF3 translation initiation complex in a signal-dependent fashion [79]. In fact, the eIF3 complex acts as a scaffolding platform that associates with mTOR and S6K1 in a growth-factor and rapamycin-sensitive manner. Under normal conditions, S6K1 is bound to the eIF3 complex, whereas mTOR association is greatly reduced. Following an activating signal, such as insulin stimulation, mTOR is recruited to the eIF3-preinitiation complex, leading to phosphorylation of the bound and inactive S6K1. Phosphorylated S6K1 is released from the eIF3 complex, and interacts with PDK1, which promotes a second phosphorylation of S6K1. Activated S6K1 then phosphorylates eIF4B, which is then recruited to the eIF3-preinitiation complex and promotes the recruitment of other initiation factors and the translation of mRNAs encoding proteins involved in muscle hypertrophy (Fig. 3, upper panel) [79]. Thus, eIF3, mTOR, and S6K1 coordinate the assembly of a translation initiation complex with enhanced translational capacity under conditions of nutrient and energy sufficiency. The understanding of how this assembly is controlled is still incomplete. However a physical and functional link exists between these proteins. Yeast two-hybrid screens have revealed that both mTOR [80] and S6K1 [79] physically interact with the f subunit of eIF3. Particularly, the inactive hypophosphorylated form of S6K1 physically associates with the Mov34 domain of eIF3f [81]. Thus, mTOR and S6K1 mediate assembly of the translation preinitiation complex through dynamic protein/protein interchange and might control the function of eIF3 [80].

### Translational regulation of eIF3f

The mRNA encoding for the eIF3f factor contains at the 5′ end a relatively short stretch of 6–12 pyrimidines, called the terminal oligopyrimidine (TOP) sequence, and consequently its mRNA is known as TOP mRNA [82]. The TOP sequence serves as a cis-acting element necessary for a growth-associated translation regulation [83]. The translational control of TOP mRNAs is used by cells to coordinate the synthesis of different ribosomal proteins, elongation factors, and several other proteins associated with the assembly or function of the translational apparatus [83]. Interestingly, among the initiation factors, only eIF3e, eIF3f and eIF3h exhibit the characteristics of TOP mRNAs [82]. A polysome mRNA analysis on HeLa, HEK293 and NIH3T3 cells has revealed that eIF3e, eIF3f and eIF3h mRNAs are mainly associated with subpolysomal particles in quiescent or growth-arrested cells, but mostly associated with polysomes in growing cells [82]. Consequently, the fact that these three eIF3 subunits are encoded by TOP mRNAs is considered relevant for their role in the growth-associated translation regulation.

It is classically known that mTOR complex 1 (mTORC1) regulates cell growth and proliferation through modulation of protein synthesis in response to growth factors, hormones and amino acids [84]. In a recent study, it has been demonstrated that the mTORC1 kinase specifically controls the translation of TOP mRNAs through the eIF4E-binding proteins (4E-BPs) and the eIF4G1 initiation factor [85]. In eIF4E-dependent initiation, mTORC1 phosphorylates the 4E-BPs promoting their dissociation from the cap-binding protein eIF4E. Active eIF4E then associates to eIF4G1, which interacts with eIF3 resulting in the assembly of the 43S pre-initiation complex on the mRNA [75]. When mTORC1 is inactivated by rapamycin or Torin1, dephosphorylated 4E-BPs bind eIF4E and thereby prevent its association with eIF4G1 and the consequent activation of TOP mRNA translation [85].

In 2010, Csibi et al. [86] reported that the decreased activity of mTORC1 kinase is correlated with the degradation of eIF3f and the accumulation of unphosphorylated forms of S6K1 during muscle atrophy. In contrast, during terminal muscle differentiation, the amount of eIF3f increases as well as mTORC1, leading to an increase in S6K1 activation and phosphorylation of rpS6 and 4E-BP1 [86]. They also observed that rapamycin treatment destabilizes the mTORC1/eIF3f interaction in differentiated myotubes, and that ablation of eIF3f in muscle cells prevents mTORC1 activity and phosphorylation of S6K1, rpS6 and 4E-BP1 [86].

Thus, in muscle cells, the eIF3f translation may be regulated in an mTORC1-dependent manner.

### Atrophy

Atrophy is a decrease in cell size mainly caused by a loss of organelles, cytoplasm and proteins. A major contribution in understanding muscle atrophy comes from the pioneering studies on gene expression profiling performed independently by Gomes and Bodine [87, 88]. The idea to compare gene expression in different models of muscle atrophy leads to the identification of a subset of genes that are commonly up- or down-regulated. Since all the diseases used for the experiments of microarray (i.e., diabetes, cancer cachexia, chronic renal failure, fasting, and denervation) have muscle atrophy in common, the up or down genes are believed to regulate the loss of muscle components and are called atrophy-related genes or atrogenes [89]. Together, these findings indicate that muscle atrophy is an active process controlled by specific signaling pathways and transcriptional programs [74]. Furthermore, the two most induced genes encode for two muscle-specific ubiquitin ligases, atrogin-1/MAFbx and MuRF1, which are upregulated in different models of muscle atrophy and are responsible for the increased protein degradation through the ubiquitin–proteasome system [87, 88]. These two genes are actually the best markers for muscle atrophy and could be considered as master genes for muscle wasting.

Ectopic expression of MAFbx in myotubes leads to atrophy and appears to be essential for accelerated muscle protein loss [90]. Interestingly, during atrophy, MAFbx physically interacts with the f subunit of eIF3 via its Mov34 domain [91], leading to its ubiquitination and its degradation by the proteasome (Fig. 3, lower panel) [90]. The silencing of MAFbx expression by small hairpin RNA interference prevents eIF3f degradation in myotubes undergoing atrophy [91]. In C2C12 myotubes that undergo atrophy, MAFbx accumulates in the nucleus and triggers the translocation of eIF3f from the cytoplasm to the nucleus. The translocation of the f subunit between the two compartments may represent a mechanism for regulating its activity during atrophy.

Interestingly, eIF3f can itself act as an enzyme by deubiquinating the transmembrane receptor Notch1 [92]. Notch 1 is involved in a highly conserved signaling pathway essential for development and is monoubiquitinated during its activation. To enter the nucleus and fulfill its transcriptional functions, this receptor needs to be deubiquitinated. The f subunit acts as a positive regulator of Notch signaling. The activated form of Notch is able to interact with eIF3f only in the presence of the E3 ubiquitin ligase Deltex [92]. It remains to be elucidated whether eIF3f works as a deubiquinating enzyme outside the translation complex or in association with the whole translation initiation complex.

### The role of eIF3f in atrophy and hypertrophy

By using an inducible protein expression system, it has been demonstrated that the genetic activation of eIF3f is sufficient to block atrophy and induces a massive hypertrophy. This cellular growth is associated with an increase of sarcomeric proteins but not the muscle regulatory factors such as MyoD and/or myogenin [91]. eIF3f is upregulated during terminal differentiation of skeletal muscle and absent in the undifferentiated embryonic rhabdomyosarcoma. Hence, in skeletal muscle, eIF3f can act as a ‘translational enhancer’ that increases the efficiency of the structural muscle protein synthesis leading to muscle hypertrophy in vitro and in vivo. These findings are consistent with previous suggestions that overload-induced hypertrophy is due to an increase in translational capacity and/or translational efficiency [93, 94]. Thus, eIF3f plays an important role in controlling muscle mass and size.

In constrast, the loss of eIF3f, due to MAFbx-mediated ubiquitination, induces atrophy in normal myotubes. Proteins destined for degradation are marked by covalent linkage with a chain of ubiquitin molecules on lysine residues for further degradation into short peptides by the 26 S proteasome. Deletion studies, focused on the identification of the lysine residues marked by MAFbx, have shown that the target for proteasomal degradation is the C-terminal domain of eIF3f. This domain contains 6 C-terminal lysine residues that serve as ubiquitination sites. The mutation of these 6 lysine into arginine residues (mutant K5-10R) does not suppress the MAFbx-eIF3f interaction nor its activity. Thus, the C-terminal domain finely regulates eIF3f activity in skeletal muscle [90]. When levels of eIF3f are maintained by electroporation of the eIF3f K5-10R mutant into adult fibers, muscles are protected from atrophy [90]. The eIF3f mutant K5-10R induces a strong hypertrophic phenotype in cellulo and in vivo, and its overexpression provokes an enhanced ability in avoiding skeletal muscle atrophy [90].

The ubiquitination of eIF3f on C-terminal lysine residues may serve two functions: addressing eIF3f degradation specifically by MAFbx during skeletal muscle atrophy and regulating the translation of specific pools of mRNAs of key sarcomeric proteins [90].

Hence, atrophy and hypertrophy processes communicate with each other via the translation regulator eIF3f [81]. Its Mov34 domain is necessary for both S6K1 and MAFbx binding. In particular, muscles undergoing atrophy accumulate the inactive hypophosphorylated form of S6K1, stimulating the MAFbx-mediated degradation of eIF3f (Fig. 3, lower panel). Whether MAFbx interacts with free eIF3f or with eIF3f molecules bound to S6K1 still remains unclear.

## Conclusion

In the light of the eIF3f properties illustrated above, a global analysis of its interactome is necessary. Two opposite features of eIF3f have emerged by analyzing the data available in the literature. This factor is able to act as both a ‘negative repressor’ of translation initiation [39] and a ‘translational enhancer’ [91], and as both a ‘downstream death executer’ in cancer cells [55] and a ‘cell growth enhancer’ in muscle cells [91]. The loss of eIF3f seems to contribute to tumorigenesis whereas its ectopic expression is sufficient to restore apoptosis in cancer cells. Interestingly, in normal myotubes, the genetic repression of eIF3f induces atrophy whereas genetic activation of eIF3f is sufficient to induce hypertrophy.

The same protein seems to act in two different ways according to the cell type considered. It is not clear whether the endogenous eIF3f protein rate is the cause or the effect of this behavior. However, a cell-type-specific role is believed to exist for eIF3f.

Several eIF3f interacting partners have been identified. During the apoptotic pathway, eIF3f is activated by CDK11p46, interacts with hnRNP K for RNA degradation, and is neutralized by Mss4. During the muscle cell growth, eIF3f interacts with mTOR/S6K1 to promote the assembly of the translation initiation complex and is degraded by MAFbx. A positive role in the activation of Notch signaling pathway has also been reported.

Moreover, two different types of eIF3 complexes (nuclear and cytoplasmic) have been found in the cell, suggesting that eIF3f could shuttle between the cytoplasm and the nucleus in a cell cycle-dependent manner.

All these data confirm that eIF3f has a multileveled control of multiple functions in the cells, outside its usual function in translation. Keeping that in mind, targeting eIF3f may be a strategy to reorganize different intracellular pathways and alter the basis of the balance between cell proliferation and apoptosis. Thus, eIF3f represents a lead candidate to use for biotherapeutic applications for both inhibiting the growth of cancer cells or muscle atrophy and, thus, preventing its progression into irreversible cachexia.
